# Applications of Sponge Iron and Effects of Organic Carbon Source on Sulfate-Reducing Ammonium Oxidation Process

**DOI:** 10.3390/ijerph19042283

**Published:** 2022-02-17

**Authors:** Yanjun Zhu, Shidong Yang, Weizhuo Wang, Lingwei Meng, Jingbo Guo

**Affiliations:** School of Civil Engineering and Architecture, Northeast Electric Power University, Jilin 132012, China; 2201900657@neepu.edu.cn (Y.Z.); wangweizhuo@neepu.edu.cn (W.W.); 20182779@neepu.edu.cn (L.M.); 20102332@neepu.edu.cn (J.G.)

**Keywords:** anaerobic ammonium oxidation (anammox), sulfate reduction, carbon source, sponge iron, microbial community structure

## Abstract

The typical characteristics of wastewater produced from seafood, chemical, textile, and paper industries are that it contains ammonia, sulfate, and a certain amount of chemical oxygen demand (COD). The sulfate-reducing ammonium oxidation process is a biochemical reaction that allows both ammonia and sulfate removal, but its low growth rate and harsh reaction conditions limit its practical application. Due to the adsorption properties of the iron sponge and its robust structure, it provides a suitable living environment for microorganisms. To reduce the negative impact on the environment, we employed 4.8 kg of sponge iron in a 2.0 dm^3^ anaerobic sequencing batch reactor (ASBR). We investigated the effects of the type and concentration of carbon sources on the performance of the sulfate-reducing ammonium oxidation (SRAO) process. The results demonstrated that during a start-up period of 90 days, the average ammonium removal efficiency and the sulfate conversion efficiency of the reactor containing the sponge iron were 4.42% and 8.37% higher than those of the reactor without the sponge iron. The addition of the sponge iron shortens the start-up time of this greenhouse gas-free denitrification process and reduces future costs in practical applications. The removal of total nitrogen (TN) significantly increased after adding organic carbon sources and then declined sharply, while the most considerable reduction of ammonium removal efficiency from 98.4% to 30.5% was observed with adding phenol. The performance of the group employing glucose as the carbon source was recovered on the 28th day, with the average ammonium removal efficiency increasing from 49.03% to 83.5%. The results of this simulation study will help the rapid start-up of SRAO in the water treatment industry and can precisely guide the application of the SRAO process for wastewater containing different organic carbon sources.

## 1. Introduction

In order to control the negative impact of climate change, many countries have reduced the emissions of greenhouse gases (GHG) [1]. China plays a crucial role in resisting climate change and has promised to reach its peak total GHG emissions by 2030 [2]. Although carbon dioxide (CO_2_) constitutes most of the greenhouse gases, other gases such as nitrous oxide (N_2_O) cause a physical effect known as radiative forcing (RF), which is the main driving force behind climate change [3]. Nitrogen removal from wastewater chiefly relies on biological processes: nitrification and denitrification. Either nitrification or denitrification inevitably produces N_2_O, the greenhouse effect of which is 265 times higher than CO_2_ [4]. Current estimates consider that humans cause 40% of N_2_O emissions, and global wastewater is the fifth largest contributor to N_2_O emissions [5] and continuously increases as the population grows and the industry develops.

The typical sulfate-reducing ammonium oxidation (SRAO) is an environmentally friendly denitrification process that converts ammonium (as the electron donor) and sulfate (as the electron acceptor) into nitrogen in an anaerobic environment, as expressed in Equation (1) [6]:

2NH_4_^+^ + SO_4_^2−^ → S + N_2_ + 4H_2_O      ΔG_0_ = −47.8 kJ/mol (1)

As autotrophic bacteria, anaerobic ammonia-oxidizing (anammox) bacteria do not generate other greenhouse gases in the anaerobic ammonium oxidation. However, the growth rate of the anammox bacteria is slow, and their nitrogen removal performance is unstable. Some studies have demonstrated that adding a 30% filling degree of zerovalent iron (ZVI) in the reactor strengthens their growth rate and stability [7]. Sponge iron is a raw material for steel making, which is made from reducing iron ore to metallic iron, easy to obtain, and inexpensive. Sponge iron is a suitable carrier for nitrogen removal and enhances biological nitrogen removal [8]. Moreover, it is a standard filler, which has the characteristics of a large specific surface area, a substantial reduction ability, and a loose and porous internal structure. Iron scraps can also stimulate both catabolism and anabolism in the presence of anaerobes [9]. 

Multiple factors, especially carbon sources, influence the coupling of ammonium oxidation and sulfur recycling. Various organic materials have different effects on the system, such as affecting sulfate reduction, denitrification, and other processes [10]. Further, the concentration of the carbon source significantly impacts nitrogen removal [11]. It has also been found that under organic conditions, a chemical oxygen demand (COD) of 160–5400 mg/L could improve heterotrophic processes, such as sulfate reduction and denitrification, thereby promoting the denitrification process [12]. A deep understanding of the influences of the species and quantities of the carbon source is essential for the nitrogen and sulfur removal processes in SRAO to enhance some biological reactions and the efficiency of nitrogen removal.

Glucose, representing fatty acids, is a well-utilized organic carbon source for sulfate-reducing bacteria (SRB) [13] that are heterotrophic [14]. As an incompletely oxidized carbon source, sodium acetate promotes the growth of both sulfate-reducing bacteria and denitrifying bacteria [15]. Phenol is chosen as the last group of organic carbon sources since it is a poisonous organic substance and has an inhibitory effect on many microorganisms; however, it is uncertain whether phenol has a negative impact on the SRAO and whether this effect is reversible. It is reported that when the concentration of the organic matter exceeds 300 mg/L, the activity of anammox declines due to the competitive inhibition between heterotrophic and autotrophic bacteria in the SRAO system [16]. Therefore, the prerequisite to ensuring the effectiveness of nitrogen removal is to control the concentration and type of organic matter in the process.

Therefore, we try to address the challenges of the practical application of SRAO by two means. First, this study develops a new method to shorten the start-up time by adding sponge iron. Second, previous studies have not been conducted on how specific carbon source types affect the SRAO system; thus, this work explores new perspectives by looking at the changes in contaminants with the addition of different organic substances to the system and trying to explain them. This paper employs 4.8 kg of sponge iron in a 2.0 dm^3^ anaerobic sequencing batch reactor to enhance the start-up stage and the simultaneous removal of nitrogen and sulfate. Three organics, namely phenol, sodium acetate, and glucose, were chosen as the typical organic sources in the range of 75–200 mg/L and added into the sludge-seeded serum bottles to investigate the effect of different organics on the SRAO process. Moreover, it aims to explain the cooperative relationships of the various bacterial species by examining the microbial community structure and investigating the influence of the organic carbon source on the microbial community by the relative abundance at a genus level. This work also supplements the application of the SRAO process to convert industrial wastewater containing nitrogen and sulfur elements and provides a reference for a similar range of applications containing anaerobic ammonia oxidation processes.

## 2. Materials and Methods

### 2.1. Sponge Iron Treatment and Synthetic Wastewater

The sponge iron was purchased from Henan Zhengjie Environmental Protection Material Company (Zhengzhou, China). The materials were sieved to collect the fractions with a particle size of 1–2 mm. The sponge iron was soaked first in a 0.5% NaOH solution for 1 h to remove the organics on its surface and then in 1 mol/L of HCl for 0.5 h to remove inorganic impurities and metal oxides on its surface. Afterward, the treated sponge iron was cleaned with distilled water until the pH of the cleaning effluent was neutral. Finally, it was rinsed with absolute ethanol three times, dried in a ventilation system, sealed, and kept for further use. To expand the specific surface area of the sponge iron as much as possible, we chose particles with a size in the range of 1–2 mm. On the basis of the reactor specifications previously reported, the volume of the sponge iron added to each reactor was 0.6 dm^3^ [17,18]; thus, the mass of the sponge iron was 4.8 kg. Inoculated sludge was added into reactors ASBR1 and ASBR2, but the sponge iron was only added into reactor ASBR2.

The synthetic wastewater contained no nitrite but did contain sulfate to donate and accept electrons. Table 1 describes the composition of the synthetic wastewater [19]. We used NH_4_Cl and K_2_SO_4_ as the primary influent substances and controlled NH_4_^+^ and SO_4_^2−^ in the range of 25.2–42.1 and 186.7–311.1 mg/L, respectively. The different carbon sources, namely phenol, sodium acetate, and glucose, were added into anaerobic serum bottles A1, A2, and A3, respectively. The chemical oxygen demand also ranged from 167 to 447 mg/L.

### 2.2. Reactor Setup and Operation

**Figure 1:**
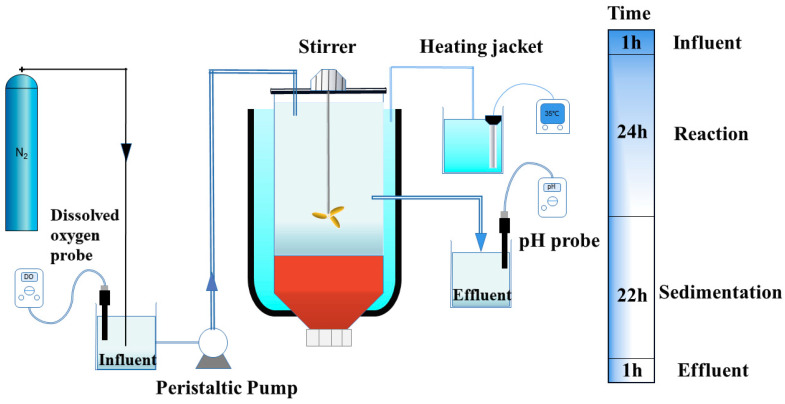
The anaerobic sequencing batch reactor and the operation time in a cycle.

Anaerobic sequencing batch reactors were employed to culture the bacteria for SRAO and investigate the effect of the sponge iron. The sludge used in the experiments was composed of the conventional anaerobic ammonium oxidation sludge preserved by the research group and the sludge from the anaerobic section of the Changchun Southeast sewage treatment plant (China). Seed sludge (0.5 L) was added into each ASBR, resulting in an initial value of the mixed liquor suspended solids (MLSS) equal to 2200 mg/L. The experiments were conducted using two sets of laboratory-scale ASBRs for sulfate-type anaerobic ammonium oxidation during the start-up process, as shown in Figure 1. The experimental setup was cylindrical with an adequate volume of 2.0 dm^3^. The reactor was equipped with an external, constant-temperature water jacket, and the water jacket compartment was 2 cm-thick and wrapped in black insulation cotton, which could protect the reactor from light and reduce heat loss to maintain a constant temperature. To control the dissolved oxygen (DO) content, we purged the influent containing the target elements by nitrogen gas before entering the reactor, and a dissolved oxygen meter measured the DO content. The ASBR reactor was operated in 48 h cycles consisting of 4 phases: an influent phase (single influent for 1 h), a reaction phase (intermittent stirring for 24 h), a sedimentation phase (22 h), and a drainage phase (1 h). The reactor received water through a peristaltic pump, and each reactor was equipped with a mechanical stirrer with a controllable speed. The stirring duration was 24 h per cycle, and the rotation speed of the stirrer was 150 rpm. Further, the whole process operated autonomously through a program controller, and the reactor temperature was set at 35 °C [20] using the water jacket. A volume of 1.0 dm^3^ of the supernatant drained per cycle was taken as the testing sample, and new synthetic wastewater was added to the system.

### 2.3. Sequencing Batch Experiments

The modified anaerobic serum bottle method was used in this work [21]. To this end, three 250 mL serum bottles were firstly sterilized, and their neck was plugged with a butyl rubber stopper for ready use. After 90 days of cultivation, the ASBR was successfully started up, and the effect of nitrogen and sulfur removal was stabilized. Each time, 200 mL of the mixture was removed from the ASBR and subjected to centrifugation at a speed of 4000 rpm for 10 min under anaerobic conditions. Next, the supernatant was removed, and the retentate was washed with an equal volume of the nutrient stock solution, followed by a second centrifugation to turn the culture into a suspension. The substrate was then added to a 250 mL serum bottle with the target components [22]. The bottles containing phenol, sodium acetate, and glucose were marked A1, A2, and A3, respectively, depending on the added organic substance. Like the ASBR reactor, after each addition of synthetic wastewater, the serum bottle was shaken in the shaker at 150 rpm for 24 h, and the supernatant was discharged after 22 h of sedimentation in one cycle. The environmental conditions were consistent with the ASBR, and 100 mL of the supernatant was extracted with a medical syringe for testing every 48 h. 

### 2.4. Measurements

The various organic masses in water were uniformly measured according to the measurement method of COD. The standard method also measured COD, NH_4_^+^, SO_4_^2−^, NO_2_^−^, and NO_3_. The mixed liquor suspended solids and the mixed liquid volatile suspended solids (MLVSS) were determined gravimetrically. The above analytical methods were derived from the fourth edition of the Water and Wastewater Monitoring and Analysis Method [23]. The TN was also calculated as the sum of the concentrations of nitrate, nitrite, and ammonium nitrogen. The dissolved oxygen and pH were measured manually by a dissolved oxygen meter (HQ30D, Hach, Loveland, CO, USA) and a pH meter (Phs-25, LiDa, Shanghai, China), respectively. The ultraviolet-visible (UV-vis) spectroscopy was conducted by a spectrophotometer model UV2400 (Hengping, Shanghai, China). Origin 8.5 performed data plotting and statistical analysis.

### 2.5. Analysis of Microbial Community 

The microbial community structures of the tested systems were analyzed via the Illumina high-throughput sequencing technology. To this end, the sludge samples were taken from the three anaerobic serum bottles of phenol, sodium acetate, and glucose at a period of 60 days and marked A1, A2, and A3, respectively. The total genomic DNA was also extracted using a PowerSoil DNA Isolation Kit (Mo Bio Laboratories, Carlsbad, CA, USA). Bacterial 16S rRNA gene fragments were amplified via the polymerase chain reaction (PCR) with the primer set 338F (ACTCCTACGGGAGGCAGCAG)/806R (GGACTACHVGGGTWTCTAAT) [24]. Majorbio Biotech Co., Ltd., Shanghai, China, analyzed the microbial community structures. The data from the measurement results were statistically analyzed using the SPSSAU platform (Qing Si Technology Ltd, Beijing, China) and computational analysis.

## 3. Results and Discussion

### 3.1. Effect of Sponge Iron during Start-Up of SRAO System

**Figure 2:**
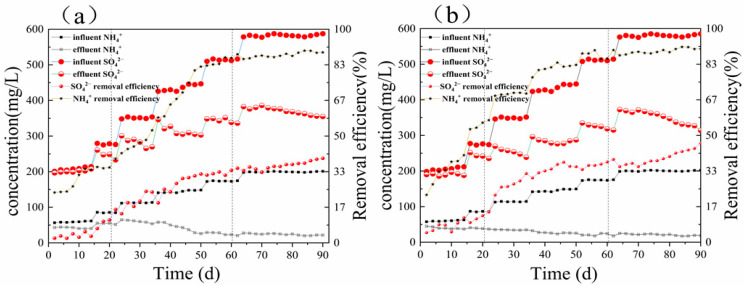
The variation in the concentration of NH_4_^+^-N, NO_2_^−^-N, NO_3_^−^-N, and SO_4_^2−^ of (**a**) reactor ASBR1 without the sponge iron and (**b**) reactor ASBR2 containing the sponge iron.

Figure 2a,b delineate the concentration of NH_4_^+^-N, NO_2_^−^-N, NO_3_^−^-N, and SO_4_^2−^ of the two anaerobic sequencing batch reactors under the conditions of adding no organic carbon source: ASBR1 without the sponge iron and ASBR2 containing the sponge iron. The functional bacteria of the system were cultured by gradually increasing the concentration of ammonium nitrogen and sulfate. In both ASBR1 and ASBR2, there was no significant nitrate and nitrite accumulation within 90 days. In the first stage (0–20 days), only a tiny amount of ammonium nitrogen and sulfate was removed, indicating that various bacteria did not proliferate in large numbers. During the second stage (from day 20 to 60), the NH_4_^+^-N concentration of the effluent changed from 55.37 to 23.24 mg/L in reactor ASBR1 without the sponge iron, and its removal percentage increased from 30% to 58%. The percentage of the SO_4_^2−^ removal also rose from 5% to 26%. Nevertheless, the NH_4_^+^-N concentration of the effluent decreased from 40.0 to 30.7 mg/L in reactor ASBR2 containing the sponge iron, and its removal efficiency enlarged from 40% to 79%. The percentage of the SO_4_^2−^ removal also increased from 8% to 28%. Furthermore, in the third stage (60–90 days), the percentage of the NH_4_^+^-N and SO_4_^2−^ removal increased from 58% to 87% and from 26% to 37%, respectively, in ASBR1 without the sponge iron and from 79% to 90% and from 34% to 41%, respectively, in ASBR2 containing the sponge iron. 

In the retardation stage of SRAO bacteria activity (0–20 days), the average removal rate of NH_4_^+^-N and SO_4_^2−^ was 40% and 8%, respectively. The nitrogen removal effect was significantly better in ASBR2 than in ASBR1. However, the treatment effect of the reactor on SO_4_^2−^ was still at a relatively low level, indicating that the sulfate-reducing anaerobic ammonia-oxidizing bacteria in the reactor have not yet shown activity, and the removal of nitrogen in the wastewater primarily relies on the joint action of nitrifying and denitrifying bacteria in the system. Further, in the process of reactor operation, some activated sludge is discharged from the reactor with the effluent, decreasing the concentration of sludge in the reactor and reducing the ammonia nitrogen removal rate. During the active performance stage (21–60 days) of sulfate-reducing anaerobic ammonia-oxidizing bacteria, reactor ASBR2 showed a significant increase in the removal of NH_4_^+^-N and SO_4_^2−^. Reactor ASBR2 presented a significant increase in the removal of NH_4_^+^-N and SO_4_^2−^, both of which increased to 79% and 28%, respectively.

Moreover, the removal effect of TN in the system gradually became better with the extension of the reactor operation time. After 60 days of continuous operation, the NH_4_^+^-N and SO_4_^2−^ in the reactor effluent significantly declined compared with the influent, indicating that sulfate-type anaerobic ammonia-oxidizing bacteria had formed in the reactor. With the extension of the reactor operation time, the dominant species in the reactor changed and was gradually replaced by sulfate-type anaerobic ammonia-oxidizing bacteria. The removal effect of both reactors for each pollutant gradually stabilized during the activity enhancement stage (60–90 days). During days 60 to 64, the concentrations of NH_4_^+^-N and SO_4_^2−^ in the reactor effluent also showed fluctuating changes with an increase in the concentration of the influent. The activity of sulfate-reducing anaerobic ammonia-oxidizing bacteria in the reactor was still gradually increasing, and the effluent water quality was slowly becoming better and stabilizing. During the operation of the reactor, nitrate did not accumulate; indeed, although nitrite occasionally accumulated in ASBR1, it did not accumulate in ASBR2, indicating that the sponge iron injection not only has a better treatment effect on NO_2_^−^-N but also promotes the initiation of sulfate-type anaerobic ammonia oxidation.

Two main factors affect the rapid start-up of the SRAO system: the low growth rate of the functional bacteria limits the rapid start-up of the SRAO [25], and the loss of the target microorganisms caused by the effluent reduces microbial enrichment. The reactor supplemented with the sponge iron lost fewer target microorganisms than the one without the sponge iron. Moreover, in the later stages of the process, the original packing formed a new biofilm in the anaerobic sequencing batch reactor with the sponge iron. When a similar weight of the seed sludge was added to both reactors, the mixed liquor suspended solids of the reactor with the sponge iron (2416 mg/L) was 15.98% higher than that of the reactor without the sponge iron (2083 mg/L) after about 150 days. Sponge iron is considered a material that can remove the organic matter in the water treatment due to its physical properties, thereby reducing the chromaticity of water [26]. The experimental results demonstrated that adding the sponge iron as a filler to the reactor could provide a suitable living environment for the sulfate anammox bacteria, perhaps due to its physical properties, and further confirmed that adding a suitable filler could enhance the growth of the sulfate anammox bacteria. 

In addition, the selection of the filler depends on many factors, including biocompatibility, availability, cost, and potential conductivity. As a nanoparticulate, iron-based, conductive material, sponge iron can directly accelerate the direct interspecies electron transfer (DIET) [27]. The DIET is a syntrophic metabolism in which free electrons flow from one cell into another through shared electrical connections without the requirement of reduced electron carriers [28]. The SRAO is also an electron transfer process, in which the electron transfer is essential for the substances participating in the reaction, where SO_4_^2−^ and NH_4_^+^ act as an electron acceptor and electron donor, respectively. In the preliminary reaction stages, when the microorganisms were not fully adapted to the influent environment, the removal rates of the ammonium nitrogen and sulfate were higher in ASBR2 than in ASBR1, which may be because the sponge iron is a conductive material and participates in the electron transfer of the SRAO system.

Moreover, the addition of the conductive material improved the resistance of the biological system to disturbances such as variations in the pH, temperature, and shock load. Comparing the removal efficiency of the two reactors revealed that the sponge iron could shorten the start-up time by enhancing the treatment effect. Therefore, it can be presumed that the microorganisms are attached to the sponge iron and undergo electron transfer.

Although the understanding of different carbon, nitrogen, and sulfur recycling systems is limited, and the low growth rate of functional bacteria is one of the main challenges affecting the practical application of the SRAO system, adding appropriate fillers may balance the complex mechanism of the SRAO and the specific environmental conditions to promote bacterial growth and enhance the nitrogen removal efficiency.

### 3.2. Influence of Organic Carbon Source in SRAO System

This paper added the sludge of the ASBR into three anaerobic serum bottles, and the biomass ratio of each serum bottle was similar to that of the anaerobic sequencing batch reactor. The influent substance, the temperature of the serum bottles, and the influent N/S were also maintained similar to those of the ASBR. The three anaerobic serum bottles reacted simultaneously for 60 days.

**Figure 3:**
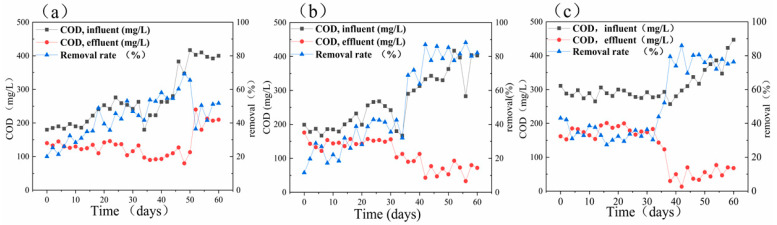
The chemical oxygen demand (COD) and the removal rate of (**a**) bottle A1 containing phenol, (**b**) bottle A2 containing sodium acetate, and (**c**) bottle A3 containing glucose.

The dissolved oxygen content of bottle A1 containing phenol ranged from 0.1 to 0.7 mg/L during 60 days of reaction. Further, the average rate of ammonium nitrogen removal was 33.16 mg/(L·day), and the efficiency of ammonium nitrogen conversion was within the range of 30.5% to 98.4% (see Figure 3a). The average rate of sulfate removal was 28.66 mg/(L·day), and the efficiency of sulfate conversion ranged from 3.7% to 37.4%. According to Figure 3a, the efficiency of COD removal also increased from 20% to 69.3%. The pH slightly declined from the initial value of 8.17 to 7.26. In the initial stages of adding the organics, the efficiency of ammonium nitrogen removal markedly improved and reached a maximum of 98.4%. In the first 14 days, the rate of COD conversion did not exceed 34%, but as the efficiency of ammonium nitrogen conversion decreased in the system, the efficiency of COD removal gradually fluctuated around 50% after 36 days. After 40 days, the efficiency of ammonium nitrogen removal began to increase. However, as the concentration of the COD of bottle A1 containing phenol increased to 400 ± 17 mg/L, the efficiency of ammonium nitrogen removal began to decline again, and this trend continued until day 60.

**Figure 4:**
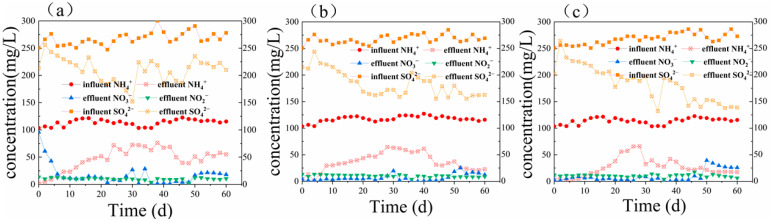
The variation in NH_4_^+^, NO_2_^−^, NO_3_^−^, and SO_4_^2−^ of (**a**) bottle A1 containing phenol, (**b**) bottle A2 containing sodium acetate, and (**c**) bottle A3 containing glucose.

When the added organic substance was sodium acetate (i.e., bottle A2), pH slightly decreased from 8.18 to 7.4 ± 0.3 after adding the synthetic wastewater, and the DO content ranged from 0.1 to 0.7 mg/L. Figure 3b demonstrates that the efficiency of COD removal increased from 11.6% on the first day to higher than 80% in the later stages of the process. Figure 4b demonstrates that the average rate of ammonium nitrogen removal was 40.29 mg/(L·day), and its average conversion efficiency was 70%. The average rate of sulfate removal was 41.78 mg/(L·day), and its average conversion efficiency gradually rose from the initial value of 14.1% to 40% ± 2% and stabilized. In the first eight days, when the organic substrate was initially added, the efficiency of ammonium nitrogen removal peaked at 98.9%, a small amount of nitrate and nitrite accumulated, and the efficiency of total nitrogen removal reached 84.2%. However, due to the change of the influent environment, the rate of nitrogen removal decreased remarkably from day 10 to day 30, and the efficiency of nitrogen removal increased steadily from 44.1% to 80% ± 2.5%.

On the contrary, the efficiency of sulfate removal improved after the addition of the organic carbon source changed the influent conditions and fluctuated with the efficiency of total nitrogen removal from day 36. From the beginning of day 40, the COD increased to 400 mg/L by raising the concentration of sodium acetate. Despite the improvement in the COD influent, the efficiency of sulfate removal was still stable at above 80%. 

In the presence of glucose as the added organic carbon source (bottle A3), although the range of variation in pH was the narrowest after the addition of the synthetic substrate, a slight decrease in pH from a mean value of 8.26 to 7.5 ± 0.18 was seen, and the dissolved oxygen content ranged from 0.1 to 0.9 mg/L. The efficiency of COD removal decreased gradually from 47.9% on day 1 to 34.4% on day 6, followed by a sudden increase to 57.9% on day 38. The efficiency of COD removal was basically stable over 80% from day 40 to day 60. Moreover, Figure 4c shows that the average rate of ammonium nitrogen removal was 43.66 mg/(L·day), and its average conversion efficiency was 76.6%. The average rate of sulfate removal was also 42.27 mg/(L·day), and its average conversion efficiency gradually increased from the initial value of 19.4% to 50% ± 1.1%. In the first 12 days, the efficiency of ammonium removal noticeably increased and was not lower than 92.03%.

Further, the efficiency of ammonium nitrogen removal followed a decreasing trend until day 30, and the efficiency of ammonium nitrogen conversion soared from 40.8% to 70.8% from day 32. However, as the influent condition stabilized, from day 50 to day 60, the efficiency of ammonium nitrogen removal again stabilized at 83.5% ± 1.5%. In contrast to the ammonium nitrogen, the overall trend of the sulfate removal was constantly increasing, from 5.6% on day 2 to 51.1% on day 60. The COD and sulfate removal efficiency tended to be stable from day 50 to day 60, but the efficiency of total nitrogen and ammonium nitrogen removal improved. 

The efficiency of pollutant removal and the activity of the various strains in the sulfate-reducing ammonium oxidation system change when the organic substance is added to the system. The organic carbon source also changes pH at a specific concentration. Heterotrophic bacteria coexisted with anaerobic ammonia-oxidizing bacteria (AAOB), improving and competing with each other under the conditions of different carbon sources in the first 10 days, but anammox was significantly inhibited during the stable period. On day 28, the activity of the denitrifying bacteria in the process using glucose as the carbon source recovered, proving that glucose was the most effective organic carbon source. Compared with nitrogen removal, sulfur removal also improved by adding organic substances under anaerobic conditions [29]. The addition of glucose raised the average conversion of sulfate from 39.5% in stage 3 of the ASBR (day 60 to day 90) to 47.5% in the stabilization period (day 50 to day 60) of group A3. In general, the efficiency of ammonium nitrogen removal was enhanced in the short term with the addition of the organic carbon source, but the activity of the denitrification-related bacteria decreased after a period. It took about 30–40 days for the system to return to its normal state. The organic utilization efficiency of the heterotrophic bacteria differed in the presence of various organic materials. Due to their metabolic types, sulfate-reducing bacteria could better use glucose and sodium acetate. The efficiency of sulfate removal and the recovery time of the nitrogen removal level of glucose were superior to those of phenol. Therefore, this work confirms that the type and dose of the organic carbon source remarkably affect the efficiency of nitrogen removal, probably due to the distinct metabolic types of the bacteria and the various organic utilization rates of the functional bacteria.

### 3.3. Analysis of Microbial Community 

**Figure 5:**
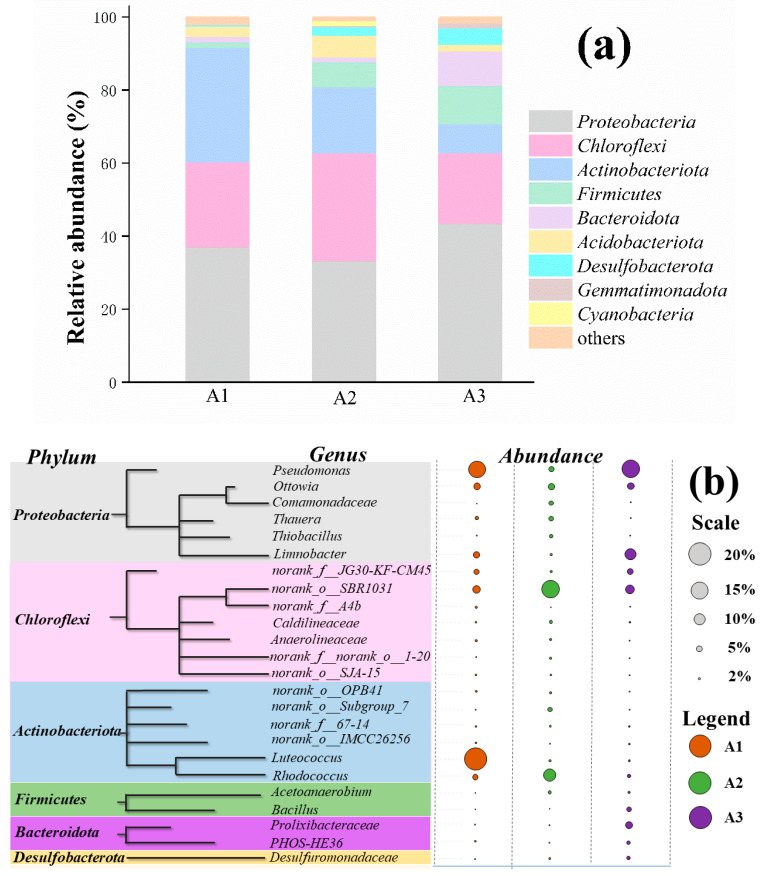
The microbial community structures of the different bacteria at a (**a**) phylum level and (**b**) genus level in the presence of three different organic carbon sources.

Figure 5a,b show the relative abundance of the major phyla and genus, respectively, in the presence of different organic carbon sources on day 60, where the legend ‘others’ includes the bacterial communities with a relative abundance of below 1%. *Proteobacteria* were the most abundant phylum in the three samples and an essential component of the microbial community. The relative abundance of *Proteobacteria* in A1 (phenol), A2 (sodium acetate), and A3 (glucose) was 37.05%, 33.17%, and 43.52%, respectively. *Proteobacteria* widely exist in anammox consortia and the nitrification reactors [30]. Some *Proteobacteria* may potentially perform SRAO (Sulfammox) [31]. Other dominant phyla were *Chloroflexi*, *Actinobacteriota*, *Firmicutes*, *Bacteroidota*, *Acidobacteriota*, *Desulfobacterota*, *Gemmatimonadota*, and *Cyanobacteria.* The proportion of *Chloroflexi* in the reactors containing phenol, sodium acetate, and glucose as the organic carbon source was 23.24%, 29.66%, and 19.31%, respectively. *Chloroflexi* have been found in seabed denitrification environments and generally exist under experimental denitrification conditions [32]. The abundance of *Actinobacteriota* in the phenol (A1), sodium acetate (A2), and glucose (A3) groups was 31.41%, 17.99%, and 7.98%, respectively. The phylum *Actinobacteriota* is generally considered an important taxon of microorganisms widely involved in sulfur reduction reactions [33], and *Firmicutes* are also capable of denitrification in heterotrophic denitrifying systems [34].

At a genus level, *Pseudomonades* were the dominant bacteria in the phenol (A1) and glucose (A3) groups, accounting for more than 15%; however, only 5.03% of the total effective sequences were in the sodium acetate group (A3), which might be due to the higher dose of the COD in group A2 than in the two other groups. In the later stages of the process, the denitrification efficiency of groups A1 and A3 was higher than that of group A2. The denitrification performance was related to *Pseudomonades,* which could be considered the nitrogen removal bacteria in the system. In addition, the typical anaerobic ammonium-oxidizing bacteria, namely *Candidatus Brocadia* and *Candidatus Kuenenia*, were present in the system under organic conditions, albeit in small proportions. *Norank_o_SBR1031* also accounted for 7.25% to 15.7% of the total sequences. This type of bacteria has been widely reported in anaerobic ammonium oxidation systems [35]. *Ottowia* (5.8% ± 0.41%) and *Thauera* (2.7% ± 1.7%) are adaptable to phenolic compounds and use aromatic or terpenoid hydrocarbons as the only carbon source [36]. The relative abundance of *Desulfuromonadaceae* in the presence of glucose (group A3) was 2.6% and 1.1% higher than that in phenol (A1) and sodium acetate (A2) groups, respectively. *Desulfuromonadaceae* is also a major genus of sulfate-reducing bacteria [37], and the rate of sulfate conversion of groups A2 and A3 was, respectively, 9.8% and 9.9% higher than that of group A1 throughout the batch experiment. *Luteococcus* also had a higher abundance in the presence of glucose, which played a potential role in the conversion of sulfate [38]. However, probably due to its possible competition with other heterotrophic bacteria for the carbon sources and the inhibitory effect of phenol, group A1 had the lowest rate of COD conversion among the three groups.

Nitrate and sulfide were rarely detected in the reactors with the different organic substrates, and nitrite occasionally accumulated; however, as the stability of the system improved, this phenomenon diminished, so it was temporary. Therefore, nitrite accumulation might be related to other biological processes involved in the system. Further, the sulfate conversion was higher under organic conditions than inorganic conditions, indicating that adding the organic carbon source enhances the heterotrophic processes, such as sulfate reduction. Therefore, the metabolic function interactions between anaerobic ammonium-oxidizing bacteria and heterotrophic bacteria are substantial [39]. 

**Figure 6:**
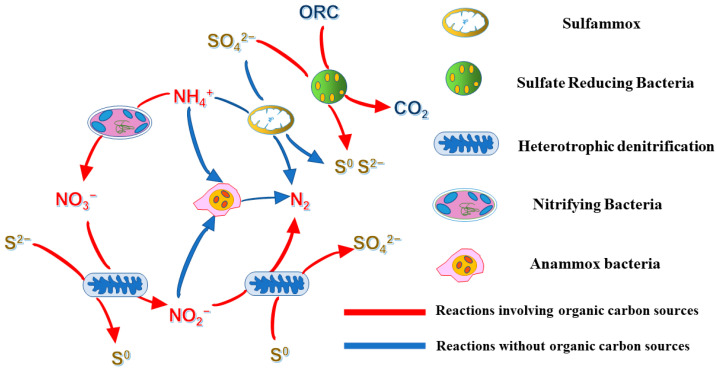
The proposed pathways of S^0^, CO_2_, and N_2_ production by the cooperation of the different bacteria.

From the analysis of the phyla and genera of the bacteria and the processes influenced by the organic carbon sources, we deduced that there is a potential relationship between the various bacteria in this work, as illustrated in Figure 6. The application of conventional nitrite-based anaerobic ammonia oxygen (ANAMMOX) technology to mainstream biological denitrification processes primarily focuses on coupled nitrification and denitrification processes. The stable conversion of ammonia nitrogen to nitrite is achieved by inhibiting the growth of nitrite-oxidizing bacteria (NOB). However, a small amount of nitrate accumulation still occurs in this process, causing great difficulty for the actual wastewater denitrification treatment process [40]. In contrast, the heterotrophic/autotrophic denitrification process can produce nitrite as an intermediate product with nitrate as a substrate, which alleviates nitrate accumulation and provides a substrate for the anaerobic ammonia oxidation–denitrification process [41]. Related studies have found that sulfide-dependent autotrophic denitrifying bacteria accumulate in the outer layer of sludge particles.

In contrast, anaerobic ammonia-oxidizing bacteria are located in the inner space of sludge particles, which may explain why sulfides can mitigate the adverse environmental effects of nitrate-nitrogen produced by the anaerobic ammonia oxidation process. Sulfur heterotrophic denitrification–anaerobic ammonia oxidation refers to the anaerobic ammonia oxidation (ANAMMOX, SRAO) reaction to degrade NH_4_^+^-N and SO_4_^2−^ in water, producing NO_3_^−^-N and HS^−^ as intermediate products, and the sulfur autotrophic denitrification reaction uses HS^−^ to reduce NO_3_^−^-N to N_2_ and produce SO_4_^2−^. This process can, on the one hand, improve the total rate of nitrogen removal and optimize the denitrification effect; on the other hand, it can circulate the elemental sulfur in the system and reduce the effect of elemental sulfur in water on the toxic effect of anaerobic ammonia-oxidizing bacteria and mass transfer efficiency. It can be inferred from the above results that the difference in the carbon source significantly impacts the heterotrophic sulfate reduction process. Furthermore, it can even proliferate other bacteria that are otherwise present in small numbers, affecting the system equilibrium.

## 4. Conclusions

If the processes related to anaerobic ammonia oxidation can be applied in practice, reducing their start-up time is currently seen as undoubtedly the best way to accelerate large-scale applications. This study developed a method to accelerate the start-up of the SRAO process using the addition of a filler to an anaerobic reactor. The addition of a sponge iron filler accelerated the growth rate of associated bacteria, resulting in a 15.98% increase in MLSS during the start-up. It also improves the removal of the target pollutant. The removal efficiency of ammonia nitrogen can be increased by 4.6% within 90 days, helping the SRAO process resist shocks in industrial wastewater treatment and enriching the theory of sustainability of nitrogenous wastewater treatment to cope with greenhouse gas emissions. Sponge iron is a means to improve the treatment effect and enrich microorganisms in the sulfate-reducing ammonium oxidation process. Although adding the different organic materials in the initial stages of the process can enhance the nitrogen removal efficiency, various organic carbon sources negatively affect the SRAO process in the subsequent stages due to the distinct types of metabolism utilized by the microorganisms. In addition, the autotrophic bacteria in the SRAO system can coexist with other heterotrophic bacteria and have the potential to simultaneously transform organic compounds, ammonium nitrogen, and sulfate.

In short, the concept of reducing greenhouse gas emissions has been accepted, and the SRAO process is an environmentally friendly way of treating wastewater to reduce greenhouse gas emissions. Efficient, economical, and environmentally friendly nitrogen and sulfur removal technologies are needed for fermentation, pharmaceuticals, and even countries that need to develop their economies. In the policy framework, adding readily available sponge iron filler into low-COD wastewater appears practical.

## Figures and Tables

**Table 1:** The composition of the synthetic wastewater.

Chemicals	Concentration (mg/L)
KH_2_PO_4_	27
CaCl_2_	180
MgSO_4_·7H_2_O	300
NaHCO_3_	1250
NH_4_Cl	125
K_2_SO_4_	444
H_3_BO_3_	0.14
MnCl_2_·4H_2_O	009.9
CuSO_4_·5H_2_O	2.5
ZnSO_4_·7H_2_O	4.3
NiCl_2_·6H_2_O	1.9
Na_2_MoO_4_·2H_2_O	2.2
CoCl_2_·6 H_2_O	24
EDTA	5
FeSO_4_	5

## Data Availability

The data used to support the findings of this study are available from the corresponding author upon request. And data archived datasets analyzed or generated during the study and presented in Figure 2, Figure 3 and Figure 4.
